# Investigation of the Differences in Antithrombin to Heparin Binding among Antithrombin Budapest 3, Basel, and Padua Mutations by Biochemical and In Silico Methods

**DOI:** 10.3390/biom11040544

**Published:** 2021-04-08

**Authors:** Réka Gindele, Krisztina Pénzes-Daku, Gábor Balogh, Judit Kállai, Réka Bogáti, Bálint Bécsi, Ferenc Erdődi, Éva Katona, Zsuzsanna Bereczky

**Affiliations:** 1 Division of Clinical Laboratory Science, Department of Laboratory Medicine, Faculty of Medicine, University of Debrecen, 4032 Debrecen, Hungary; gindele.reka@med.unideb.hu (R.G.); kpenzes@med.unideb.hu (K.P.-D.); balogh.gabor@med.unideb.hu (G.B.); kallai.judit@med.unideb.hu (J.K.); bogati.reka@med.unideb.hu (R.B.); ekatona@med.unideb.hu (É.K.); 2 Department of Medical Chemistry, Faculty of Medicine, University of Debrecen, 4032 Debrecen, Hungary; bbalint@med.unideb.hu (B.B.); erdodi@med.unideb.hu (F.E.)

**Keywords:** antithrombin, heparin-binding site, Antithrombin Budapest 3, Antithrombin Basel, Antithrombin Padua, surface plasmon resonance, molecular modelling

## Abstract

Antithrombin (AT) is a serine protease inhibitor, its activity is highly accelerated by heparin. Mutations at the heparin-binding region lead to functional defect, type II heparin-binding site (IIHBS) AT deficiency. The aim of this study was to investigate and compare the molecular background of AT Budapest 3 (p.Leu131Phe, ATBp3), AT Basel (p.Pro73Leu), and AT Padua (p.Arg79His) mutations. Advanced in silico methods and heparin-binding studies of recombinant AT proteins using surface plasmon resonance method were used. Crossed immunoelectrophoresis and Differential Scanning Fluorimetry (NanoDSF) were performed in plasma samples. Heparin affinity of AT Padua was the lowest (KD = 1.08 × 10^−6^ M) and had the most severe consequences affecting the allosteric pathways of activation, moreover significant destabilizing effects on AT were also observed. KD values for AT Basel, ATBp3 and wild-type AT were 7.64 × 10^−7^ M, 2.15 × 10^−8^ M and 6.4 × 10^−10^ M, respectively. Heparin-binding of AT Basel was slower, however once the complex was formed the mutation had only minor effect on the secondary and tertiary structures. Allosteric activation of ATBp3 was altered, moreover decreased thermostability in ATBp3 homozygous plasma and increased fluctuations in multiple regions of ATBp3 were observed by in silico methods suggesting the presence of a quantitative component in the pathogenicity of this mutation due to molecular instability.

## 1. Introduction

Antithrombin (AT) is a single-chain glycoprotein belonging to the serpin family, synthesized in the liver, the molecular weight of it is 58,200 Da [1]. After the cleavage of the propeptide (32 amino acids), the mature protein is composed of 432 amino acids. Two glycoforms of AT are present in the circulation, the majority as α-glycoform (90–95%). β-glycoform is present to a lesser extent (<10%). In the presence of heparin or heparin-like substances the inhibitory effect of AT to serine protease clotting factors is highly accelerated.

The first case of AT deficiency was described by Egeberg et al. in 1965 and the first functional AT defect, named as AT Budapest, was reported by Sas et al. in 1974 [2,3]. AT deficiency is classified into type I (quantitative) and type II (qualitative) [4]. In type II deficiency, the defect may involve the reactive site (IIRS), the heparin-binding site (IIHBS) or it can exert a pleiotropic effect (IIPE) [5].

Individuals with inherited AT deficiency have an increased risk for thromboembolic events, including different types of venous and arterial thrombosis [6,7,8,9,10,11,12,13]. AT deficiency subtypes, however, do not necessarily have the same clinical phenotype, as it has been reported in different patients’ populations. In addition, even within the same subtype, there may be phenotypic differences that are characteristic of each mutation. In a French study involving the highest number of patients the risk of venous thromboembolism (VTE) was lower in IIHBS as compared to type I [14]. IIHBS AT deficiency associated with arterial thrombotic events (ATE) more often than type I, and other type II deficiencies [15]. Type I was more frequent and more severe than type II AT deficiency in a Japanese study [16]. Type I and IIRS types were more severe than IIHBS Basel in a small study from Denmark [17]. Within type I AT deficiency null mutations associated with more severe symptoms than other mutation types [15,18]. Interestingly, within IIHBS AT deficiency clinical symptoms show significant heterogeneity. In a study from Finland, where the prevalence of AT Basel (p.Pro73Leu) is relatively high, this type associated with high thrombosis risk; moreover, ATE and pregnancy complications were also common [19]. VTE was frequent in two HBS AT deficiency subtypes, AT Basel and AT Padua (p.Arg79His), and ATE was associated with these types more often than in type I [20]. In our clinical study involving 246 AT deficient cases, 75% of them were IIHBS patients with heterogeneous clinical phenotype [21]. As AT Budapest 3 (p.Leu131Phe) is a founder mutation in Hungary, we had the possibility to investigate high number of patients with this type and to compare their clinical phenotype to other IIHBS types [22]. While AT Budapest 3 (ATBp3) homozygosity represented by far the most severe thrombophilia (even more severe than type I) and associated with VTE, AT Basel rather associated with ATE and AT Padua showed more pregnancy complications. In selected ATE patients ATBp3 heterozygosity was observed [23].

Laboratory diagnosis of AT deficiency is based on a functional test in which the inhibitory effect of AT on active factor X (FXa) or thrombin (FIIa) is investigated in the presence of heparin [1]. It has been observed that the currently available functional tests in certain AT deficiency subtypes have different sensitivity, which makes the laboratory diagnosis difficult. It was suggested that in the case of the AT Cambridge II (p.Ala416Ser), a IIRS mutation, which is relatively common, primarily in the UK AT deficient population, the FXa-based assays are not sufficiently sensitive [24,25]. In the contrary, FXa-based tests in the presence of heparin (hc-anti-FXa) in general are more sensitive to IIHBS AT deficiencies as compared it to FIIa-based tests [26]. When the assay is performed in the absence of heparin, a progressive activity of AT is detected (p-anti-FXa). Ratio of p-anti-FXa to hc-anti-FXa is markedly increased in IIHBS AT deficiency [27]. It was shown, that in type IIHBS patients hc-anti-FXa were different according to the specific mutations [21]. While all assays gave abnormal results in ATBp3 homozygosity, ATBp3 heterozygotes, AT Basel and AT Padua heterozygous mutants were detected with different sensitivity in different commercially available assays suggesting that not only the enzyme (i.e., FIIa or FXa) but also other assay conditions, as heparin concentration and ionic strength were responsible for the heterogeneous results [19,20,21,28]. Assays with high heparin concentration showed lower sensitivity to AT Basel and AT Padua than to ATBp3 suggesting differences in the strength of AT-heparin binding and in the activation of AT among these mutants. Moreover, antigenic concentration of ATBp3 samples, which is usually lower than AT antigen of AT Basel or AT Padua samples suggest more complex consequence of this mutation than being a heparin-binding defect.

To investigate, whether the features of heparin binding of AT are different in case of different IIHBS mutants, in silico methods offer a possibility. Several molecular dynamics-based (MD) studies related to AT have been published. However, only a few publications are available where AT mutations were investigated, including one from our research group involving the AT Debrecen variant (p.Leu205Pro) [29]. To the best of our knowledge, there has been no long molecular dynamics simulations applied to study the consequences of AT IIHBS variants, as yet. The ATBp3 variant was investigated only by using Web-based structure analysis tools, but not MD [30].

The aim of the present study was to investigate the molecular background of the differences among type IIHBS AT variants focusing on their heparin-binding affinity.

## 2. Materials and Methods

### 2.1. Antithrombin Deficient Patients and Their Routine Laboratory Investigation

Between January 2007 and August 2020, a total of 329 non-related AT deficient patients (index patients) and their family members (total n = 446) were diagnosed at the Clinical Center of the University of Debrecen. Blood samples were collected into 0.109 M citrated vacutainer tubes (Beckton Dickinson, Franklin Lakes, NJ, USA) and plasma samples were stored at −80 °C until analysis. AT activity was determined by chromogenic assay based on factor Xa-inhibition in the presence of heparin (Innovance AT, Siemens, Marburg, Germany) and also in the absence of heparin (progressive AT activity, measured by Labexpert Antithrombin H+P, Labexpert Ltd., Debrecen, Hungary) according to the manufacturers’ instructions on Siemens BCS-XP coagulometer. The AT antigen (AT:Ag) concentration was measured by immunonephelometry (Siemens, N Antiserum to Human Antithrombin III).

### 2.2. Mutation Analysis of Antithrombin Deficient Patients

Genomic DNA was isolated from peripheral whole blood using QIAamp DNA Blood Mini kit (Qiagen GmbH, Hilden, Germany). Sanger sequencing was executed to identify mutations in the exons, the flanking intronic regions and in the promoter of *SERPINC1* gene using an ABI3130 Genetic Analyzer and Sequencing Analysis 5.4 software (Thermo Fisher Scientific, Carlsbad, CA, USA). If Sanger sequencing did not find causative mutations, multiplex ligation-dependent probe amplification (MLPA) was performed using SALSA MLPA KIT P227 (MRC-Holland, Amsterdam, the Netherlands) using an ABI3130 Genetic Analyzer. The MLPA products were analyzed by GeneMapper Software 4.1 (Thermo Fisher Scientific).

### 2.3. In Vitro Expression of Wild Type and Mutant Antithrombins

The cDNA clone ORF-NM_000488_pcDNA3.1(+) wild type AT (WT) was purchased from ImaGenes GmbH (Berlin, Germany). ATBp3, AT Basel and AT Padua mutant plasmids were created by us using the QuickChange Site-Directed Mutagenesis (Agilent Technologies, Santa Clara, CA, USA) kit according to manufacturer’s instructions.

Stable transfection of Human embryonic kidney (HEK-293) cells with huSERPINC1_pcDNA3.1(+) WT, ATBp3, AT Basel, and AT Padua plasmids was performed with Lipofectamine^®^3000 Transfection Kit (Invitrogen, Carlsbad, CA, USA). Geneticin^®^ Selective Antibiotic (Gibco, Thermo Fisher Scientific, Carlsbad, CA, USA) was used as a selective agent, the optimal Geneticin concentration of the culture media was 400 µg/mL. Cells were grown at 37 °C and 5% CO_2_ in a humidified incubator. Media containing expressed WT and mutant AT (ATBp3, AT Basel and AT Padua) proteins were harvested and concentrated on an Amicon^®^ Ultra 30 kDa (Merck Millipore, Burlington, VT, USA) column for further investigations.

### 2.4. Preparation of Antithrombin from In Vitro Expressed Recombinant Antithrombins and from Normal and ATBp3 Homozygous Plasma by Affinity Chromatography

AT proteins (WT, ATBp3, AT Basel, and AT Padua) were purified from the concentrated culture media of transfected HEK-293 cells by affinity-chromatography using Goat anti-human Antithrombin IgG (Affinity Biologicals, Ancaster, ON, Canada) that was covalently coupled to Sepharose 4B gel. The concentration of purified AT proteins was determined by immunonephelometry (Siemens, Marburg, Germany). Normal and ATBp3 homozygous plasma samples were also purified by this protocol.

### 2.5. Crossed Immunoelectrophoresis

Crossed immunoelectrophoresis (CIE) was performed according to Sas et al. [31]. The first electrophoresis was performed at 150 V for 60 min in 1% agarose containing 16.3 U/mL of unfractionated sodium heparin. The second dimension electrophoresis was performed at 100 V for 180 min in 1% agarose containing 1% rabbit anti-human AT antiserum (Sigma, Saint Louis, MO, USA).

### 2.6. Surface Plasmon Resonance

Surface plasmon resonance (SPR) assays were performed on a Biacore 3000 instrument (GE Healthcare, Uppsala, Sweden). For assaying the binding characteristics of different AT mutants to heparin, heparin SPR sensorchip (Heparin Approx. 50 nm hydrogel chip, XanTec bioanalytics GmbH, Dusseldorf, Germany) was used. One of the advantages of the device used is that it can divide the sensor chip into 4 cells. We were, therefore, able to test all the four recombinant (WT and 3 mutant) AT proteins on the same chip. A total of 90 μL of each AT mutants, diluted in running buffer (HEPES 10 mM, NaCl 150 mM, EDTA 3 mM, surfactant 0.005% (*v*/*v*), pH 8.4), was injected into the microflow cell in 6 different concentrations (50, 100, 150, 300, 500, and 750 nM). The flow rate was 10 μL/min. Between two measurements chips were regenerated with 30 μL regeneration buffer (10 mM glycin-HCl, pH 2.5, GE healthcare, Uppsala, Sweden). Langmuir 1:1 binding model was used for curve fitting. From the sensorgrams, the association and dissociation rate constants (k_a_ and k_d_), and the equilibrium association and dissociation constants (K_A_ and K_D_) were calculated by the BIAevaluation software (GE Healthcare, Uppsala, Sweden). Optimal pH of the reaction was previously determined (pH 8.4) and the reaction proceeded at this pH [27].

### 2.7. NanoDSF

NanoDSF is a differential scanning fluorimetry method used for accurate analysis of protein folding and stability. NanoDSF measures the intrinsic tryptophan or tyrosine fluorescence for the analysis. During unfolding of proteins tryptophan becomes hydrated and its fluorescence intensity maximum is shifted from 330 nm to 350 nm. The thermal stability of a protein can be described by the thermal unfolding transition midpoint (T_m_) at which half of the protein population is unfolded. Thermal stability is also characterized by the onset temperature (T_onset_) of the denaturation. T_m_ is determined from the inflection point of the denaturation curve, which is the ratio of the tryptophan fluorescence at 330 and 350 nm plotted against the temperature or from the maximum of its first derivative. The dual wavelength system of the Prometheus NT.48 (NanoTemper Technologies GmbH, Munich, Germany) was used to characterize the thermal unfolding processes of wild type and Budapest3 mutant form of AT protein. The samples were loaded into standard glass capillaries at a concentration of 8 μM in triplicate. The sample volume was 10 µL per capillary. During the analysis, the samples were heated from 20 °C to 95 °C at a ramp rate of 1 °C/min. T_m_ and T_onset_ values were determined using PR.ThermControl v2.1.2 software (NanoTemper Technologies GmbH, Munich, Germany) using first derivative analysis of 350 nm/330 nm fluorescence ratio plotted against the temperature. The T_m_ values were determined from 3 independent experiments.

### 2.8. In Silico Methods

To investigate the effects of the mutations on the AT heparin pentasaccharide binding, as well as the stability of the protein, we have built two types of model systems. First, the 1T1F X-ray diffraction structure was used for the system not containing the pentasaccharide, representing the “non-activated” state [32]. Second, the pentasaccharide-bound, activated AT was modelled starting from the X-ray structure 1NQ9 [33]. We have constructed models corresponding to both activation states for WT AT, as well as the three mutants, ATBp3, AT Basel, and AT Padua. The latter variant was simulated with both neutral and protonated His79 (His47 in the mature protein). All systems were solvated using the CHARMM variant of the TIP3P water model. The simulation boxes were cubic and the minimum distances between the protein (or the ligand) and the edges of the box were 12 Å. Na^+^ and Cl^−^ ions were added to set ionic strength to 0.15 M.

Similarly to our previous paper regarding the AT—pentasaccharide binding [34], we have chosen the CHARMM36m force field for the AT protein and the CHARMM carbohydrate FF for the pentasaccharide [35,36]. Like β-AT, the protein was glycosylated on three Asn residues: Asn-96, -155, and -192 in the mature protein [37]. Due to their large size, the oligosaccharide chains were truncated in the same way as in the article mentioned previously. We used the CHARMM-GUI web server to generate the topology files for all three systems [38,39,40].

Thus the appropriate sampling of such conformational transitions often requires advanced sampling techniques. The Gaussian Accelerated Molecular Dynamics (GAMD) method was chosen because it offers significantly enhanced sampling while it does not depend on pre-defined reaction coordinates [41].

The AMBER 16 pmemd.cuda software was used for the molecular dynamics (MD) and GAMD simulations [42], (ambermd.org). All model systems were subjected first to two consecutive energy minimizations, 2000 steps each. The first minimization included 500 steps using the steepest descent and 1500 steps of conjugate gradient method, with the protein and the ligand position restrained. In the following 2000 steps, no residues were restrained, and the conjugate gradient method was applied. The system was then heated from 0 K to 310 K in a 2 ns MD simulation, with all non-solvent and non-ion atoms restrained. The heating was followed by 2 ns of pressure equilibration.

A dual-boost scheme was used in our GAMD simulations [41]. The sigma0P and sigma0D parameters for the GAMD simulation were set to their default values (6.0). All GAMD simulations included a 60 ns equilibration phase before the “production” part. In the first 10 ns, no GAMD potential was applied on the system and the 4–10 ns part was used for data collection. In the next 50 ns the GAMD potential was applied on the system. The GAMD parameters were updated at regular intervals except for the first 5 ns of the 50 ns phase.

The production GAMD simulations were performed on 310 K under NVT conditions. The Langevin thermostat was applied in the simulation, with a gamma constant of 2.0. In the production simulations the Monte Carlo barostat was used for pressure coupling. Long-range electrostatic interactions were computed using the PME method [43]. A Coulomb cutoff of 12 Å was used in the simulations. A force switch was applied between 10 Å and 12 Å for the Lennard-Jones interactions, recommended for CHARMM force fields. All production simulations were 600 ns long. For each mutant, two parallel simulations were run for the 1T1F-based and three for the 1NQ9-based systems. This corresponds to combined simulation times of 1.2 µs and 1.8 µs, respectively, for each AT mutation. The total production simulation time in our work was 15 µs.

The CPPTRAJ [44] software was used for most trajectory analysis, including RMSD and RMSF calculations as well as clustering. “Generalized correlation” calculations were performed using a method developed by Lange and Grubmüller, based on an earlier work by Ichiye and Karplus [43,45].

## 3. Results

### 3.1. Clinical and Laboratory Characteristics of Patients with Antithrombin Type II Heparin-Binding Site Mutations

Until recently, we have diagnosed *n* = 19 individuals with AT Basel, *n* = 31 individuals with AT Padua. Most of our patients, however, carried the ATBp3 mutation and both homozygotes (*n* = 52) and heterozygotes (*n* = 239) have been found (Table 1). Venous thrombosis was registered in almost all ATBp3 homozygotes (88.8%) and it was also relatively frequent in ATBp3 heterozygotes, while a great proportion of AT Basel was free from venous thrombotic symptoms at the time of data collection. A few patients in the ATBp3 and AT Padua groups suffered from pulmonary embolism without having obvious embolic source, as investigated by standard imaging methods (i.e., complete compression ultrasonography and contrast venography). Arterial thrombosis was the most frequent in AT Basel. Patients with arterial thrombosis were investigated by cardiac ultrasound to exclude the presence of patent foramen ovale. The frequency of pregnancy complications, as pre-term birth or spontaneous abortions was slightly higher in AT Padua patients; however, the difference was not statistically significant. Median of hc-anti-FXa AT activity was obviously the lowest in ATBp3 homozygotes and the Innovance AT method, which was used for the measurements, gave AT activity results below the cut-off value in all AT Basel and Padua mutations. There were a few ATBp3 heterozygpus patients (*n* = 4) with hc-anti-FXa AT activity slightly above the cut off value of 80%. The p-anti-FXa AT activity in the absence of heparin in the reagent was significantly lower in both heterozygous and homozygous ATBp3 mutants as compared to AT Basel and AT Padua. Ratio of p-anti-FXa AT to hc-anti-FXa AT activity was the highest in ATBp3 homozygotes demonstrating the largest effect of this genotype on AT-heparin interaction. AT antigen concentration was significantly lower in the ATBp3 groups when comparing it to the other two mutants.

**Figure 1:**
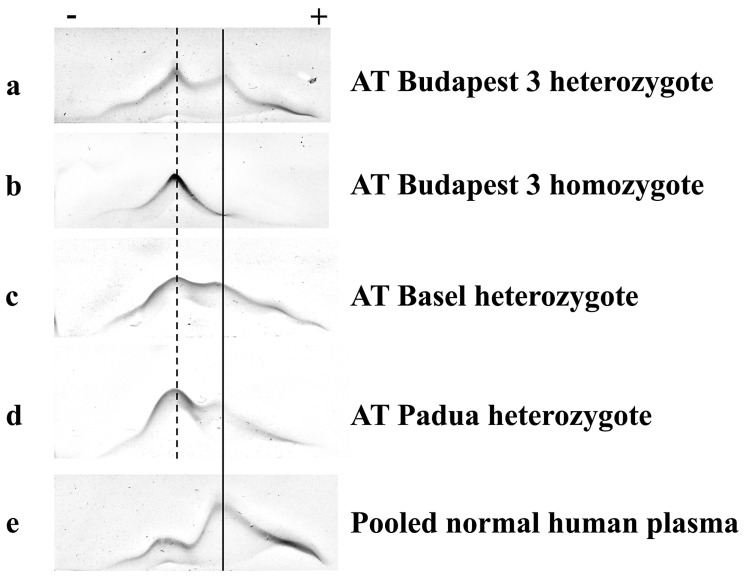
Crossed immunoelectrophoresis of normal and AT mutant plasmas. (**a**) AT Budapest 3 heterozygote; (**b**) AT Budapest 3 homozygote; (**c**) AT Basel heterozygote; (**d**) AT Padua heterozygote; (**e**) pooled normal human plasma. The first dimension, with heparin, runs from left to right.

The crossed immunoelectrophoresis (CIE) results in the plasma of patients with different mutations demonstrated a heparin low affinity fraction (dashed line) and a heparin high affinity fraction (continuous line) in all of the heterozygote plasmas (Figure 1a,c,d). In the case of ATBp3 homozygote plasma only heparin low affinity AT could be visualized (Figure 1b).

The less anodal abnormal peak in the AT Padua heterozygote plasma was larger than the normal peak (Figure 1d). This phenomenon is concordant with the previous results of Girolami et al. [46].

### 3.2. Thermostability of Wild Type and Budapest 3 Homozygous Antithrombin

**Figure 2:**
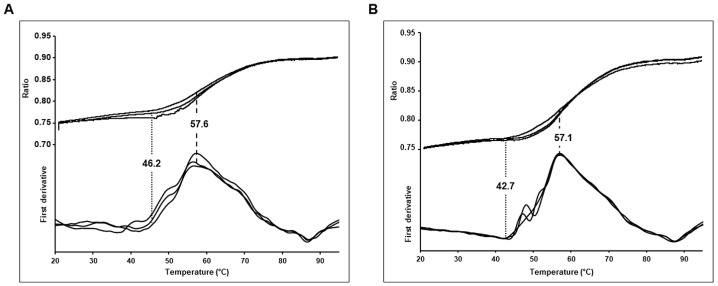
Melting curves for wild type (**A**) and ATBp3 mutant (**B**) antithrombin proteins obtained by nanoDSF technique. The stability parameters of the proteins were determined at 8 μM concentration in triplicate by Prometheus NT.48 device. Dotted lines indicate the onset of denaturation (T_onset_), dashed lines indicate melting point (T_m_). An apparent difference can be observed in both the T_m_ (Δ0.5 °C) and T_onset_ (Δ3.5 °C) values for the wild type and mutant protein, which demonstrates the difference between their thermal stability. As the T_m_ occurs at a lower temperature (57.1 °C) in case of ATBp3 mutant, therefore its thermal stability is suggested to be lower than that of wild type AT.

In nano DSF (Differential Scanning Fluorimetry) experiments comparing the ATBp3 homozygous mutant and WT AT separated from human plasma (*n* = 3 independent measurements) we observed significant differences (Figure 2). Change in ratio of tryptophan fluorescence at 350 nm to 330 nm with respect to increase in temperature was used to characterize the thermal stability of WT and ATBp3 mutant AT proteins. Two parameters, the onset temperature (T_onset_) of denaturation and the transition midpoint (T_m_) were determined using the melting curves of the proteins. In the case of the WT AT the T_onset_ and T_m_ values were 46.2 ± 1.3 °C and 57.6 ± 0.1 °C, respectively, while for the mutant AT the T_onset_ was 42.7 ± 1.47 °C and the T_m_ was 57.1 ± 0.03 °C. Both T_m_ (*p* = 0.0031) and T_onset_ (*p* = 0.0371) values were significantly lower in case of the ATBp3 mutant compared to WT AT indicating a lower thermostability for the mutant protein.

### 3.3. Investigation of Heparin-Binding Characteristics of Wild Type and Different IIHBS Antithrombin Mutants

**Figure 3:**
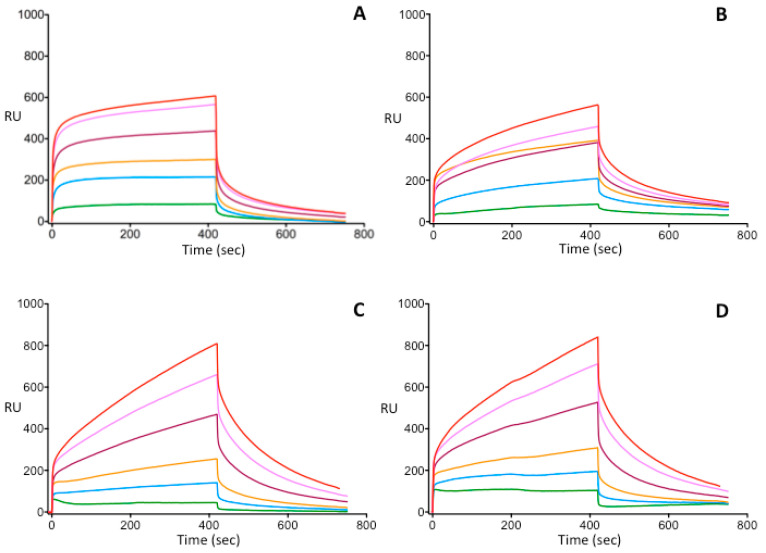
Analysis of antithrombin–heparin interaction of wild type and mutant antithrombin proteins by Surface Plasmon Resonance method. Wild type and mutant AT proteins were purified from the media of transfected HEK293 cells by affinity chromatography. AT forms at six different concentrations (1, 3, 5, 7, 9, and 11 nM from bottom to top curves in the pictures) were injected into the 4 different microflow cells on the same chip. Sensorgrams corresponding to the wild type and three different mutant AT forms are shown on panels (**A**–**D**): (**A**) wild type AT (**B**) AT Budapest 3 (**C**) AT Basel (**D**) AT Padua. SPR sensorgrams demonstrate the binding of the given AT to heparin at different concentrations. RU, response unit.

As for AT Basel and AT Padua only heterozygote patients exist (homozygosity is suggested to be lethal) we investigated the heparin-binding characteristics of different type IIHBS mutants and WT AT in a purified system using recombinant proteins. On Figure 3, sensorgrams corresponding to the wild type and three different mutant AT-s obtained in SPR experiments are demonstrated. Parameters calculated from the sensorgrams obtained at six different AT concentrations were averaged for each AT. (Figure 3). As it was expected, the strongest AT-heparin binding was observed in the presence of the WT AT protein (K_D_ = 6.4 × 10^−10^ M, K_A_ = 2.2 × 10^9^ 1/M). The association rate constant (k_a_) was the highest (k_a_ = 1.37 × 10^7^ 1/Ms) for WT AT among the investigated purified recombinant AT proteins. These data suggest that the formation of the AT-heparin complex occurs most rapidly in case of WT AT. The dissociation rate constants in all mutants were of the same order of magnitude (10^−3^). (For the WT AT k_d_ = 6.75 × 10^−3^ 1/s).

Out of the mutant AT proteins, ATBp3 formed the most stable complex with heparin (K_D_ = 2.15 × 10^−8^ M, K_A_ = 1.62 × 10^8^ 1/M) as well as complex formation was the fastest (k_a_ = 3.25 × 10^5^ 1/Ms). The dissociation rate constant for ATBp3 mutant was k_d_ = 2.47 × 10^−3^ 1/s. As compared to the data obtained for the WT protein, ATBp3 appeared to exhibit a significantly weaker interaction with heparin, with a slower association rate. SPR studies performed on WT and ATBp3 homozygous mutant proteins isolated from human plasma also showed approximately two orders of magnitude differences in K_D_ values (data not shown).

For the AT Basel mutant, the association/dissociation kinetic and equilibrium parameters were as follows: k_a_ = 1.03 × 10^4^ 1/Ms and k_d_ = 4.45 × 10^−3^ 1/s; K_D_ = 7.64 × 10^−7^ M and, K_A_ = 2.40 × 10^6^ 1/M. These data show that AT Basel binds to heparin more slowly than WT AT and ATBp3 and the association constant of AT Basel is almost 1 order of magnitude lower than that of ATBp3.

Among all mutants, AT Padua had the weakest interaction with heparin (K_D_ = 1.08 × 10^−6^ M, K_A_ = 2.37 × 10^6^ 1/M) and it had the slowest complex formation (k_a_ = 1.01 × 10^4^ 1/Ms). However, the dissociation rate constant (4.51 × 10^−3^ 1/s) was almost equal to the k_d_ values obtained for the other mutants.

### 3.4. In Silico Modeling of Wild Type and Different Type IIHBS Mutant Antithrombin Proteins

#### 3.4.1. Conformation of the 22–46 Loop

The 22–46 loop, close to the heparin binding site, has been suggested to play a role in the control of AT pentasaccharide binding [47]. This loop is highly flexible, and many of its amino acids are not resolved in X-ray diffraction structures. Despite the lack of experimental data, enhanced sampling MD simulations allowed us to study the conformation of this loop. As molecular modeling was performed using the structure of the mature protein the numbering in the sections of in silico experiments follows the classical way, i.e., the first amino acid in the mature protein is numbered as +1. (ATBp3 corresponds to Leu99Phe, AT Basel corresponds to Pro41Leu and AT Padua corresponds to Arg47His.)

Among the amino acids affected by the mutations studied, Pro41 (position of AT Basel) is located in the 22–46 loop; thus, the conformation of this loop may be an important factor in the reduced heparin affinity of this variant.

**Figure 4:**
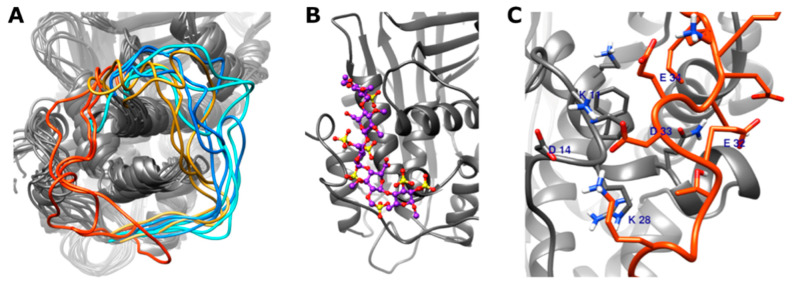
In silico simulations of the 22–46 loop of antithrombin. (**A**) Superposition of representative frames from the two wild type (WT) and the two Pro41Leu simulations without pentasaccharide, obtained by clustering, three conformations from each trajectory. The 22–46 loop is shown in yellow and orange for the Pro41Leu and in two different shades of blue for the WT simulations. (**B**) The predicted binding position of idraparinux, a pentasaccharide for comparison. Structure was obtained by the modification of pentasaccharide in X-ray diffraction structure 1NQ9 (see details in the text) and energy minimization. (**C**) Interacting amino acids between amino acids of the 22–46 loop and the pentasaccharide-binding region of the protein, in one of the representative frames from the Pro41Leu system. The representative frames were selected using K-means clustering, as implemented in the CPPTRAJ software [44].

In the two simulations of the non-activated AT Basel variant (Pro41Leu), two major types of conformations were observed. In the first type, the loop occupied a position very close to the heparin-binding site, forming salt bridges and hydrogen bonds with the amino acids of the site (Figure 4). In the second type, mostly observed in the other 600 ns trajectory, the distance from the heparin-binding region was large. This suggests a significantly increased conformational variability of the loop compared to the WT, with some conformations potentially interfering with the binding of a heparin pentasaccharide.

However, in the pentasaccharide-bound AT simulations, no close contact was observed between the loop and the heparin-binding region. This suggests that the altered conformation of this loop probably has no or only small destabilizing effects on the pentasaccharide binding once a “strong” complex has been formed. The highly charged nature of the pentasaccharide and the altered electrostatic effects could prevent the loop from transitioning into the alternative conformation.

#### 3.4.2. DSSP (Define Secondary Structure of Proteins) Analysis of the N-Terminal Part of the Antithrombin Protein

We analyzed the changes of secondary structure in the N-terminal-part (amino acids 1–145, this includes the entire heparin binding site, but not the “A” beta sheet) using the DSSP method [45], as implemented in the CPPTRAJ software [44]. This analysis was performed for both the “not activated AT” (1T1F) and “AT-pentasaccharide complex” (1NQ9) systems (Supplementary Figures S1 and S2).

Regarding the 1T1F-based systems, conformational changes were evident in the AT Basel simulations in the region described in the previous section as compared to the WT AT. However, no significant conformational changes were visible in the heparin-binding regions, including helix P (amino acids 112–120) (For the discussion of helix P conformation in GAMD simulations, see reference [48].) Among the “complex” simulations, the AT Padua (Arg47His) variant (and especially its neutral from) showed conformational changes in the 30–35 region. Similarly to the 1T1F-based simulations, there were no significant conformation changes in the heparin-binding region. Some elongation of helix D was visible in the ATBp3 (Leu99Phe) trajectories as compared to the wild type.

#### 3.4.3. Root Mean Square Fluctuations Analysis

Root mean square fluctuations (RMSF) are common analysis methods to describe the flexibility of amino acids in a protein. It can be used for measuring the increases in fluctuation caused by a missense mutation. Additionally, conformational changes can cause increases or decreases in the fluctuations at specific parts of the molecule.

**Figure 5:**
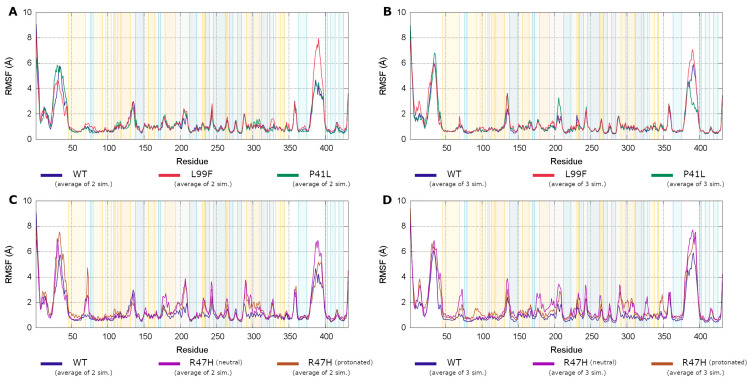
Root mean square fluctuations (RMSF) from our Gaussian Accelerated Molecular Dynamics (GAMD) trajectories. Averages from two (for the 1T1F, non-activated) or three (1NQ9, AT-pentasaccharide complex) independent simulations are shown. Parts (**A**,**C**) correspond to the non-activated AT-simulations, while (**B**,**D**) to the AT-pentasaccharide complex trajectories. The background of the plots is colored by the secondary structure of the region (orange—α-helix, blue—β-sheet.). The RMSF values for individual trajectories are shown in Supplementary Figures S3 and S4. Regions with higher fluctuation in the 3D structure of AT are depicted in Supplementary Figures S5 and S6.

Among the “not activated” AT simulations, the ATBp3 variant showed moderate increases in fluctuations as compared to the wild type protein and the AT Basel variant (Figure 5A). We could observe increased fluctuations both in parts of the protein close to the affected amino acid (residues 50–100), as well as in more distant regions (amino acids 320–330, 360–380, and 420–430). However, the increases in fluctuations are minor in other parts of the tertiary structure. These findings are consistent with a variant in which the stability of the native conformation was moderately decreased, but still secreted in considerable amounts. In case of the AT Basel variant, increases in fluctuation was mainly observed in the 110–130 and 300–320 regions. The most interesting finding, however, was the highly increased fluctuations in the AT Padua variant (Figure 5C). This increase was observed in both protonation states of residue 47. The increases are particularly large in the region close to the F helix (180–210) and in the helices in the 290–310 region. This suggests that this variant may affect the conformation and also the allostery in distant parts of the molecule.

Concerning the heparin-activated state, the RMSF values are expected to reflect the change in the stability of the tertiary structure, similarly to the non-activated state. However, conformation changes in the regions with higher-than-average fluctuations, known to be involved in the allosteric mechanism, can also trigger an increase in the RMSF. Regions that participate in this process include the C-terminal end of the D helix, the reactive center loop and the exosite interacting with FXa and FIXa. The ATBp3 variant showed increases in fluctuations compared to the WT, similarly to the “non-bound” state. The C-terminal end of helix D (amino acids 125–135) plays an important role in the allosteric activation. In case of the WT system, significant decrease was observed for the “bound” state as compared to the 1T1F-based simulations of non-activated WT AT (Figure 5B). However, this was not the case for ATBp3 and AT Basel variants; the RMSF values were still high in both states (Figure 5B,D). In case of the AT Padua variant the increased fluctuations, which were observed in the 1T1F-based systems representing non-bound AT, could be observed also in the heparin-bound simulations of this variant. The regions with the largest increases are nearly the same in the two types of systems for AT Padua mutation (Figure 5D).

#### 3.4.4. Analysis of Allosteric Pathways

The conformational activation of AT is essentially an allosteric process in which the heparin-binding site, the reactive center loop, and the coagulation factor binding exosites are involved. Like in our previous work, the allosteric pathways were investigated using a method developed by Lange and Grubmüller [49], which can detect correlated motions in MD trajectories. We performed this analysis for both the 1T1F, and the 1NQ9-based systems, and the results are shown in Supplementary Figures S7 and S8.

In general, the AT Basel variant showed similar patterns to the WT AT, indicating that this mutation probably has only relatively small effects on allostery. As a contrast the patterns were different in case of the ATBp3 and especially the AT Padua mutations. This likely corresponds to significant alterations in the allosteric processes.

#### 3.4.5. Root Mean Square Deviations of Pentasaccharide Binding

The Root Mean Square Deviations (RMSDs) of the pentasaccharide ring and interglycosidic atoms compared to the X-ray diffraction structure were used to describe the conformation of the ligand. There was no significant difference between the pentasaccharide RMSD values observed in the WT, AT Basel, and ATBp3 simulations (Supplementary Figure S9). In contrast, the AT Padua protonated variant showed increased pentasaccharide RMSD values as compared to all other simulations. Surprisingly, this decrease in the binding strength could only be observed in the protonated variant.

Full or nearly full dissociation of the ligand was not observed in any of the simulations for a certain time period. It should be noted, however that all variants investigated had a glycosylation pattern corresponding to β-AT that likely resulted in a stronger AT-heparin interaction. The reason for using β-AT in the simulations was the difficulty of the proper conformational sampling of a large oligosaccharide in α-AT close to the binding site. Very extensive MD simulation would be required to get a proper “average” for the interaction between the glycan and the pentasaccharide.

## 4. Discussion

The recognition of type IIHBS AT deficiency is dated back to the eighties when several observations on abnormal AT-heparin interaction were published [50,51,52,53,54]. AT Basel was associated with arterial thrombosis in the early report of Brennan, and the mutant AT showed an abnormal peak in heparin-Sepharose affinity chromatography [51]. In a monoclonal antibody-based heparin-binding affinity assay AT Basel demonstrated 40-fold reduction in heparin-binding affinity comparing it to normal AT [52]. AT Padua showed 30-fold decrease in heparin affinity in another study using other method for investigation [53]. The ATBp3 variant showed reduced heparin affinity by heparin-Sepharose chromatography, and the antiproteinase activity was decreased as compared to normal AT in the presence of unfractionated heparin (UFH) or the AT-binding pentasaccharide [55]. According to the results of an elegant study of Martinez-Martinez et al. plasma samples of ATBp3 homozygous patients showed an increased fraction of AT with low heparin affinity [54]. The major finding of that study was the demonstration of the compensatory role of β-AT in the type IIHBS mutants ATBp3 and AT Basel. They did not investigate AT Padua, however in case of an Arg to Cys mutation in the same position the recombinant β-isoform could compensate for the strong effects of this mutation in the interaction with heparin. Direct comparison of the heparin-binding features of ATBp3, AT Basel, and AT Padua by using uniform methodology for all mutants, however, has not performed, as yet. As AT Basel and AT Padua homozygous patients do not exist, only recombinant, purified systems yielding high amount of homozygous mutant AT variants are able to serve as basis of biochemical studies. Complex in silico studies comparing the behavior of these type IIHBS mutants have not been executed, either.

In our relatively large AT deficient patient group (n = 449 patients), the type IIHBS subtype is the most common. We demonstrated that the frequent occurrence of the ATBp3 mutation is the result of a founder effect in this AT deficient population [22]. Several clinical studies suggested differences in the clinical and laboratory phenotypes, especially in the strength of AT-heparin binding among the different mutants resulting in type IIHBS AT deficiency [14,21,56,57]. The type IIHBS ATBp3, AT Padua, and AT Basel mutations were identified in association with VTE, ATE and pregnancy complications as well.

The laboratory characteristics of our IIHBS AT deficient patients were heterogeneous. The hc-anti-FXa AT activity was decreased. The p-anti-FXa AT activity was low normal or it was in the normal range. The AT antigen levels were normal in AT Basel and in AT Padua patients. In the case of ATBp3 homozygous and heterozygous individuals, however, the AT antigen concentration was significantly lower upon comparison it to that of AT Basel and AT Padua. This suggests mild quantitative defect (besides heparin-binding abnormality) in ATBp3 mutation, which may be caused by protein instability. According to the results of our nanoDSF experiments, homozygous ATBp3 purified from plasma exerted decreased thermostability as compared to WT AT. (As no homozygous AT Basel and AT Padua plasmas were available this experiment was not done in these mutations) ATBp3 instability was also demonstrated by our in silico studies, where elongation of helix D was observed in its native form and increased RMSF was found in its non-activated state. These findings, however, do not seem to be deleterious but may lead to mild secretion defect or may cause increased elimination of ATBp3 AT due to its instability. According to our knowledge no pulse-chase experiments have been performed to confirm these hypotheses to date, however in two studies mutations in AT gene close to the ATBp3 position led to decreased secretion [58,59].

In this study we focused on the AT-heparin binding characteristics of ATBp3, AT Basel and AT Padua mutants and intended to explore the background of differences in their heparin-binding affinity.

Pooled normal human plasma and AT deficient plasma from ATBp3 heterozygous, ATBp3 homozygous, AT Basel heterozygous and AT Padua heterozygous patients were examined by CIE. As it was visualized on the electrophoresis the heparin binding affinity in the case of ATBp3 heterozygous and AT Basel heterozygous plasmas did not differ as much. By examining AT Padua heterozygous plasma a stronger low heparin affinity fraction was observed, while in ATBp3 homozygous plasma only this low heparin affinity fraction appeared. These observations suggested AT Padua as the most severely affected AT mutant from the point of view of heparin affinity.

Due to the lack of AT Basel and AT Padua homozygous subjects a direct comparison of the biochemical consequences of these mutations in plasma samples is problematic. We therefore established recombinant models for further investigations. The recombinant WT AT and ATBp3, AT Basel, AT Padua proteins were expressed in HEK293 cells. The stable expressed proteins were harvested, concentrated, and purified by affinity chromatography. These purified proteins were examined by SPR. We investigated the binding affinities of WT and mutant AT to immobilised heparin surface. SPR has several advantages among methods used for testing molecular interactions. It allows label-free, real-time, medium-throughput tests and requires only a small amount of materials and reagents. It uses an optical method to measure the change in refractive index of the medium close to the gold surface to monitor the binding of analyte molecules to ligand molecules, which are immobilized on the metal surface [60]. Only a very few studies have been published in which AT-heparin interaction was investigated by SPR technique, as yet. Moreover no study exists with a direct comparison of type IIHBS mutants. A novel method has been developed for the easy measurement of heparin’s anticoagulant activity using SPR by Zhao et al. [61]. The anticoagulant activity of target heparin was evaluated by measuring the competitive AT binding of analyte heparin in the solution phase and USP heparin immobilized on chip surface. Heparins, obtained from different animal sources, and low molecular weight heparins were analyzed. The results were reproducible and correlated well with the results of chromogenic assays (correlation coefficient r = 0.98 for anti-Xa and r = 0.94 for anti-IIa) used in routine hemostasis laboratories. This well designed competition SPR method has been previously used in many studies for characterizing heparin-protein interaction [62,63,64]. Biomolecular interaction analysis using SPR was utilized to record and analyze prothrombin, thrombin, AT, and fibrinogen heparin binding properties in real time [65]. Biotinylated heparin, heparin–albumin conjugate, and albumin were immobilized onto streptavidin-coated sensors as ligands, respectively. The binding pattern of AT to both heparin and heparin–albumin conjugate, although specific, was biphasic, possibly due to a conformational change during the binding process. Steady-state kinetic analysis revealed a *K*_D_ value of 281 ± 24 × 10^−9^ M for the heparin surface. For the conjugate surface, a *K*_D_ of 53 ± 5 × 10^−9^ M was calculated, indicating a higher affinity toward heparin–albumin conjugate. (The results for the other proteins mentioned in the paper are not discussed here.) As compared to our results, these K_D_ values are closest to the K_D_ values obtained for our recombinant WT AT (K_D_ = 6.4 × 10^−10^ M). In our experiments AT Padua had the lowest heparin affinity (K_D_ = 1.08 × 10^−6^ M) and K_D_ value for ATBp3 mutant AT (K_D_ = 2.15 × 10^−8^ M) was the closest to that of WT AT, however there was still a two-magnitude difference. Heparin affinity of AT Basel was in between (K_D_ = 7.64 × 10^−7^ M).

We intended to explain the differences observed in the biochemical studies by means of in silico analysis of WT AT and mutant AT proteins. In the simulations of AT Basel variant without a ligand, we could observe a novel conformation of the 22–46 loop, which can probably interfere with the binding of the pentasaccharide. Among the variants studied, AT Basel mutation affects the fluctuations and allosteric pathways the least as compared to the WT AT, therefore it is unlikely that this mutation has strong destabilizing effect on the secondary and tertiary structures. For the ATBp3 variant, we could observe increased fluctuations in the protein, both close to the heparin-binding site as well as in more distant regions. From the “generalized correlation” calculations, we can conclude that this variant likely affects the allosteric pathways that are involved in the conformational activation of AT. According to our simulations, the AT Padua variant has the most severe consequences. The fluctuations as measured by the RMSF of α-carbon atoms were significantly increased comparing it to the normal AT both in the non-activated and activated states. Based on the “generalized correlation” analysis, the allosteric pathways were also affected. This variant seems to have destabilizing effects in addition to the heparin-binding defect. However, we could not observe significant dissociation of the pentasaccharide in any of the AT-pentasaccharide complex simulations. From the simulation trajectories, we were able to draw qualitative conclusions on the pentasaccharide binding of WT and mutant AT. However, the insufficient conformational sampling of partially or fully dissociated conformations did not allow us to calculate free energies from these data. Such calculations would require a different enhanced sampling technique, for example the recently published LiGaMD method [66].

It is difficult to translate the findings obtained in the biochemical analyses of purified systems and those observed in the in silico analyses directly to clinical consequences due to several reasons. First, in vitro studies were executed in recombinant purified homozygous AT variants, while patients with different mutations (excluding ATBp3 homozygotes) are heterozygotes, thus clinical phenotype are obviously influenced by AT expressed from their normal allele. Ratio of α-AT to β-AT may also be different from patient to patient influencing heparin-binding affinity. Second, as thrombosis is a common complex disease the risk conferred by AT deficiency is definitely modified by different genetic and environmental factors which also different among patients. Moreover there may be protective factors (not fully understood as yet), which modify the clinical severity in AT deficiency by gene–gene or by gene–environment interactions.

## 5. Conclusions

According to our results there may be different molecular mechanisms in the background of altered AT-heparin interaction in case of type IIHBS AT mutations. AT Padua mutation has the strongest effect on AT. We observed the slowest AT-heparin complex formation and the weakest interaction with heparin. AT Padua also exerted a larger low-affinity fraction than normal fraction in CIE experiments. Molecular modeling studies well explain these findings, as AT Padua showed conformational changes in the N-terminal 30–35 region of AT and highly increased fluctuations in RMSF analysis suggesting that the mutation affects both the conformation and the allostery of distant parts of AT Padua molecule. AT Basel binds to heparin more slowly than other mutants, presumably due to the conformational change in the 22–46 loop. Once AT-heparin complex is formed the allosteric activation of AT Basel and the stability of AT Basel-heparin complex might be only slightly affected. ATBp3 showed the fastest and strongest AT-heparin complex formation in SPR studies, however the allosteric activation of ATBp3 is affected, moreover the increased fluctuation in multiple regions of the molecule suggested that this variant has a destabilizing effect. These findings together with the decreased thermostability of homozygous ATBp3 isolated from human plasma suggest the presence of a quantitative component in the pathogenicity of this mutation.

## Figures and Tables

**Table 1:** Clinical and laboratory characteristics of type II heparin-binding site (IIHBS) antithrombin (AT) deficient patients.

	AT Basel	AT Padua	ATBp3 Homozygous	ATBp3 Heterozygous	*p* Value
	*n* = 19	*n* = 31	*n* = 52	*n* = 239	
Male/female	6/13	8/23	25/27	98/141	NS
Heparin cofactor anti-FXa AT activity (%)	58 (44–74)	57 (40–70)	17 (9–53) +	57 (36–86)	<0.001
Progressive anti-FXa AT activity (%)	100 (73–120)	106 (73–126)	73 (56–100) +	85 (60–228) ++	<0.001
p-anti-FXa to hc-anti-FXa ratio	1.77 (1.25–2.05)	1.84 (1.28–2.10)	4.30 (2.06–8.40) +	1.50 (0.94–3.12) ++	<0.001
AT antigen (g/L)	0.30 (0.25–0.36)	0.30 (0.24–0.35)	0.21 (0.13–0.29) +	0.24 (0.14–0.35) ++	<0.001
Frequency of patients with VTE %	11.1	26.9	88.8 ^§^	42.1	<0.001
Frequency of patients with PE %	0	11.5	6.7	6.3	NS
Frequency of patients with ATE %	44.4 ^§§^	7.7	6.7	7.6	0.002
Frequency of females with pregnancy complications %	37.5	42.9	38.9	35.4	NS

AT activity and antigen values and p-anti-FXa to hc-anti-FXa ratio are presented as median (min–max). Reference intervals for hc-anti-FXa activity, p-anti-FXa activity and AT antigen are 80–120%, 82–118%, and 0.19–0.31 g/L, respectively. VTE = venous thromboembolism, PE = pulmonary embolism, ATE = arterial thromboembolism. + Heparin cofactor anti-FXa AT activity (hc-anti-FXa), progressive anti-FXa AT activity (p-anti-FXa), AT antigen and the ratio of p-anti-FXa activity to hc-anti-FXa activity of ATBp3 homozygotes were significantly different from all other types in multiple comparisons using Kruskal–Wallis test with Bonferroni correction. The hc-anti-FXa AT activity values of ATBp3, AT Padua, and AT Basel were statistically not different. ++ The p-anti-FXa AT activity, AT antigen and the ratio of p-anti-FXa activity to hc-anti-FXa activity of ATBp3 heterozygotes were significantly different from the corresponding values of AT Basel and Padua. ^§^ Frequency of VTE in ATBp3 homozygotes was significantly higher as compared to all other groups. ^§§^ Frequency of ATE in AT Basel was significantly higher as compared to all other groups.

## Data Availability

The datasets generated for this study are available on request to the corresponding author.

## Supplementary Materials

The following are available online at https://www.mdpi.com/article/10.3390/biom11040544/s1, Figure S1: Root mean square fluctuations of the α-carbon atoms, from the simulations not containing a pentasaccharide ligand (“1T1F-based”). Figure S2: Root mean square fluctuations of the α-carbon atoms, from the simulations of AT-pentasaccharide complex (“1NQ9-based”). Figure S3: The “generalized correlation” matrices calculated using a method by Lange and Grubmüller, for the simulations not containing a pentasaccharide ligand (“1T1F-based”). Figure S4: The “generalized correlation” matrices calculated using a method by Lange and Grubmüller, for the simulations of AT-pentasaccharide complex (“1NQ9-based”). Figure S5: Root mean square fluctuations (RMSF) of the α-carbon atoms in the simulations not containing a pentasaccharide ligand (“1T1F-based”). Figure S6: Root mean square fluctuations (RMSF) of the α-carbon atoms in the simulations of AT-pentasaccharide complex (“1NQ9-based”). Figure S7: Analysis of the secondary structure in region 1-145 (which includes helices P and D) using the DSSP method, from the simulations not containing the pentasaccharide ligand. Figure S8: Analysis of the secondary structure in region 1-145 (which includes helices P and D) using the DSSP method, from the AT-pentasaccharide complex simulations. Figure S9: RMSD of the ring and interglycosidic atoms in the heparin pentasaccharide from all AT-pentasaccharide system simulations, compared to the X-ray diffraction structure 1NQ9.
