# Supplementary material for: Investigation of the Differences in Antithrombin to Heparin Binding among Antithrombin Budapest 3, Basel, and Padua Mutations by Biochemical and In Silico Methods
Source: Biomolecules. 2021 Apr 8;11(4):544. doi: 10.3390/biom11040544 (PMC8068293; doi:10.3390/biom11040544)
Supplement: Supplementary file 1 [file biomolecules-11-00544-s001.pdf]

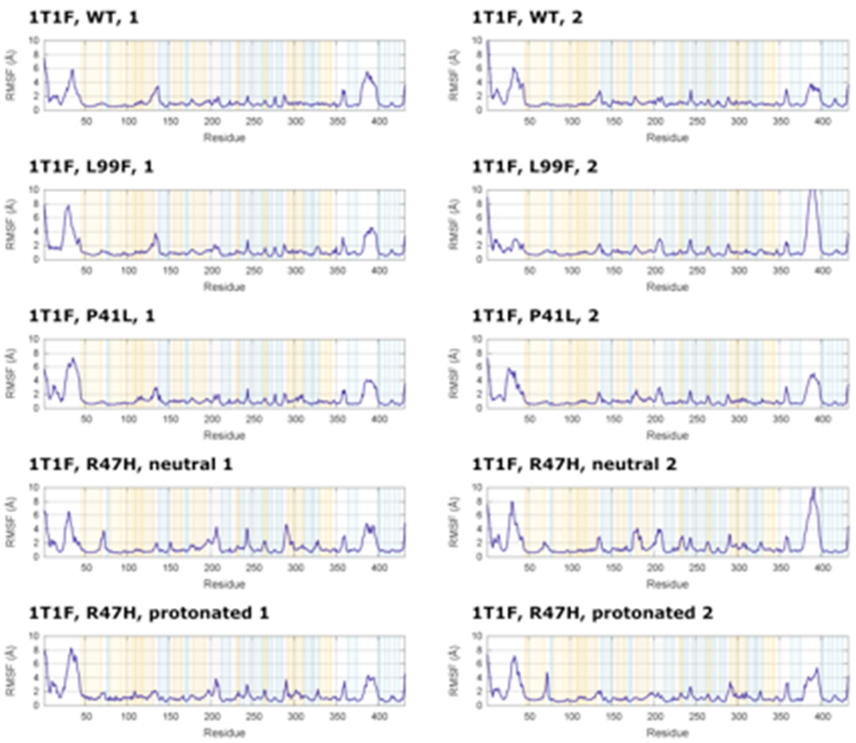

Supplementary figure 1: Root mean square fluctuations of the α-carbon atoms, from the simulations not containing a pentasaccharide ligand ("1T1F-based"). The background of the plots is colored by the secondary structure of the region (orange – α-helix, blue – β-sheet.)

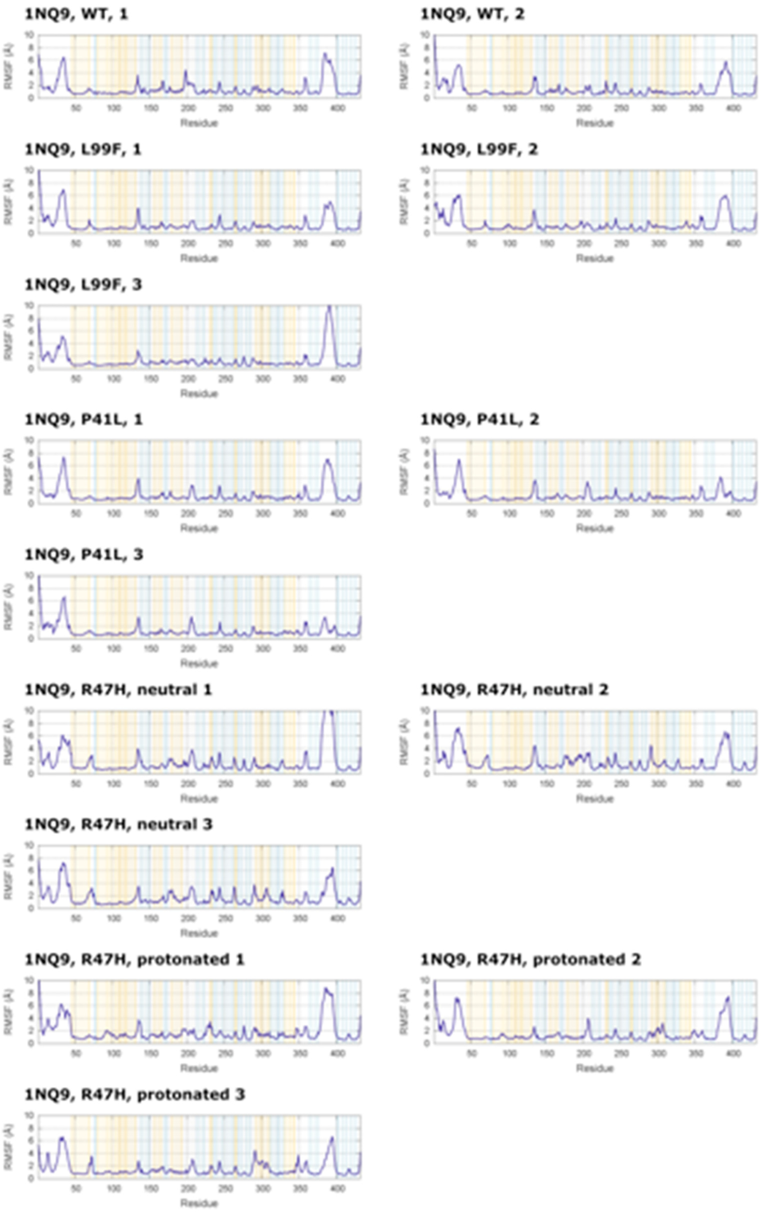

Supplementary figure 2: Root mean square fluctuations of the α-carbon atoms, from the simulations of AT-pentasaccharide complex ("1NQ9-based"). The background of the plots is colored by the secondary structure of the region (orange – α-helix, blue – β-sheet.)

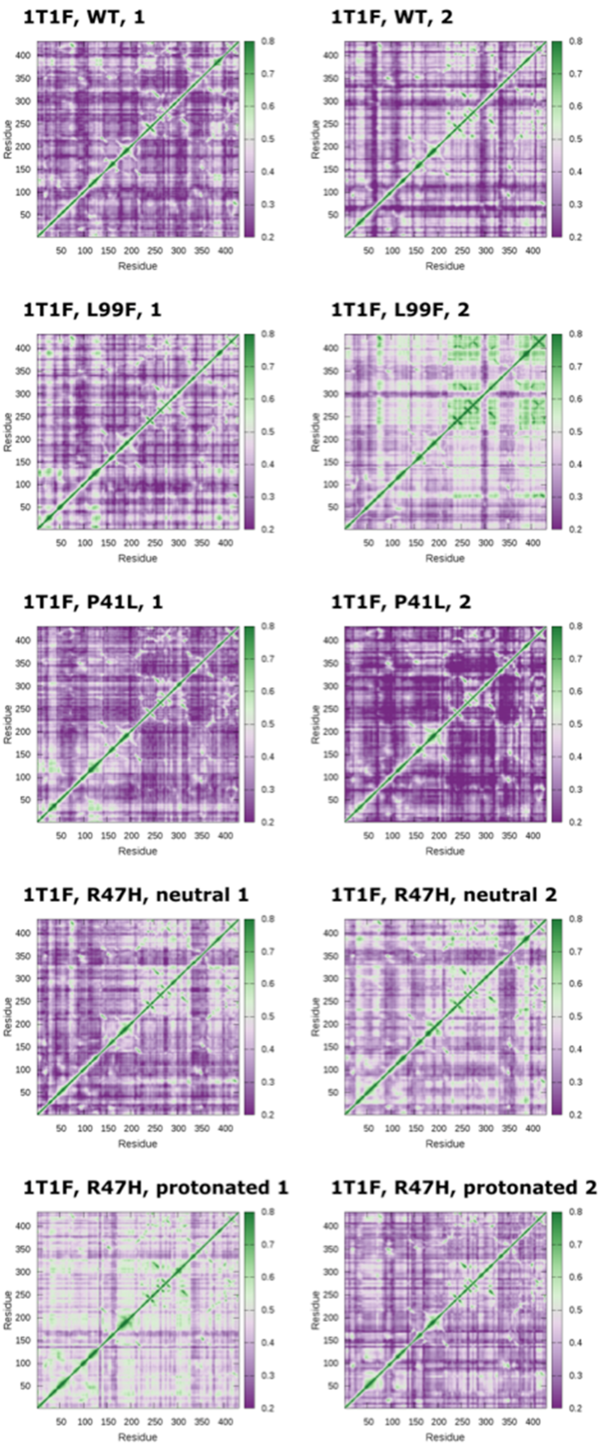

Supplementary figure 3: The “generalized correlation” matrices calculated using a method by Lange and Grubmüller, for the simulations not containing a pentasaccharide ligand (“1T1F-based”). Such matrices contain information about the allosteric pathways.

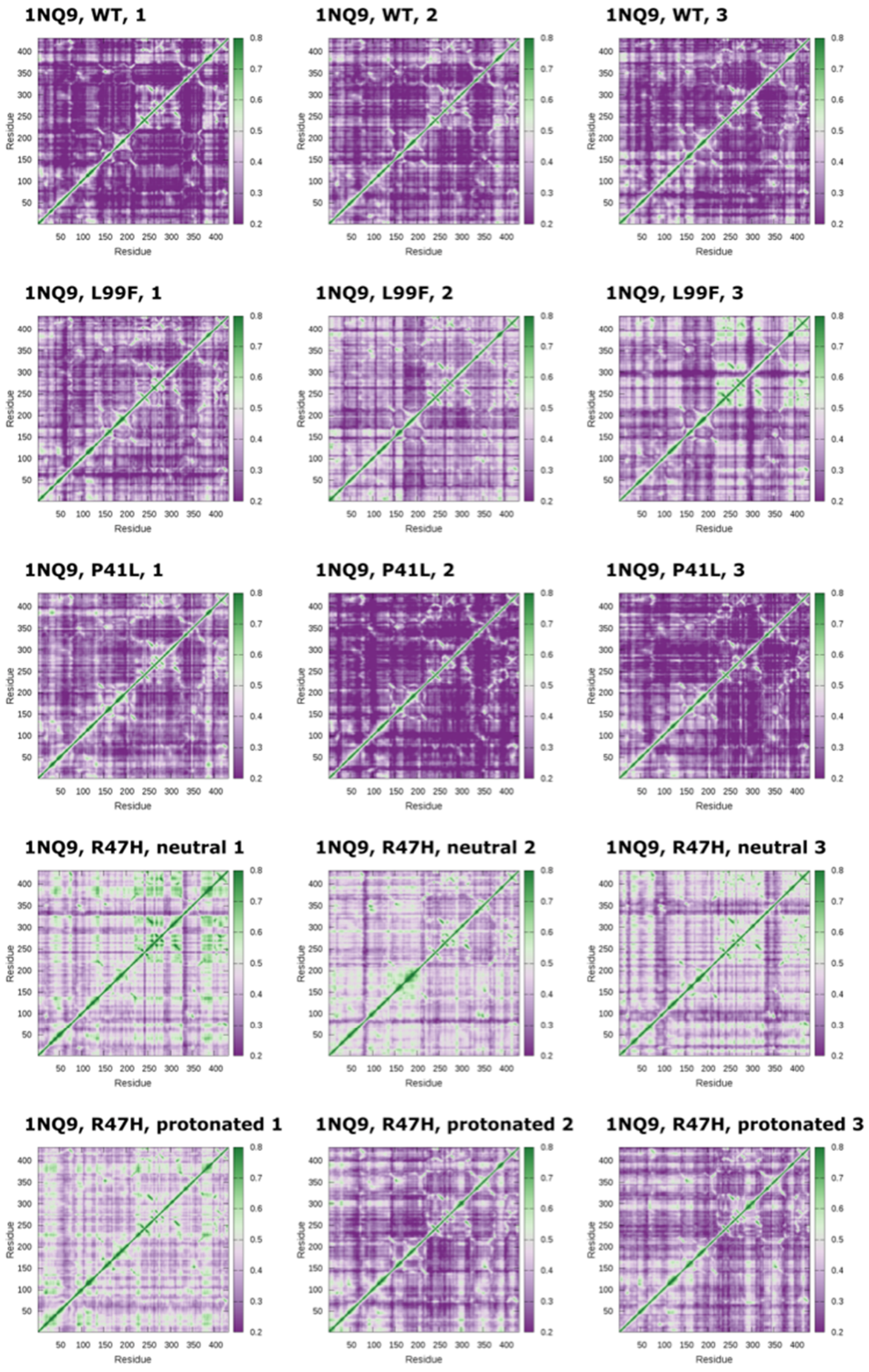

Supplementary figure 4: The “generalized correlation” matrices calculated using a method by Lange and Grubmüller, for the simulations of AT-pentasaccharide complex (“1NQ9-based”). Such matrices contain information about the allosteric pathways.

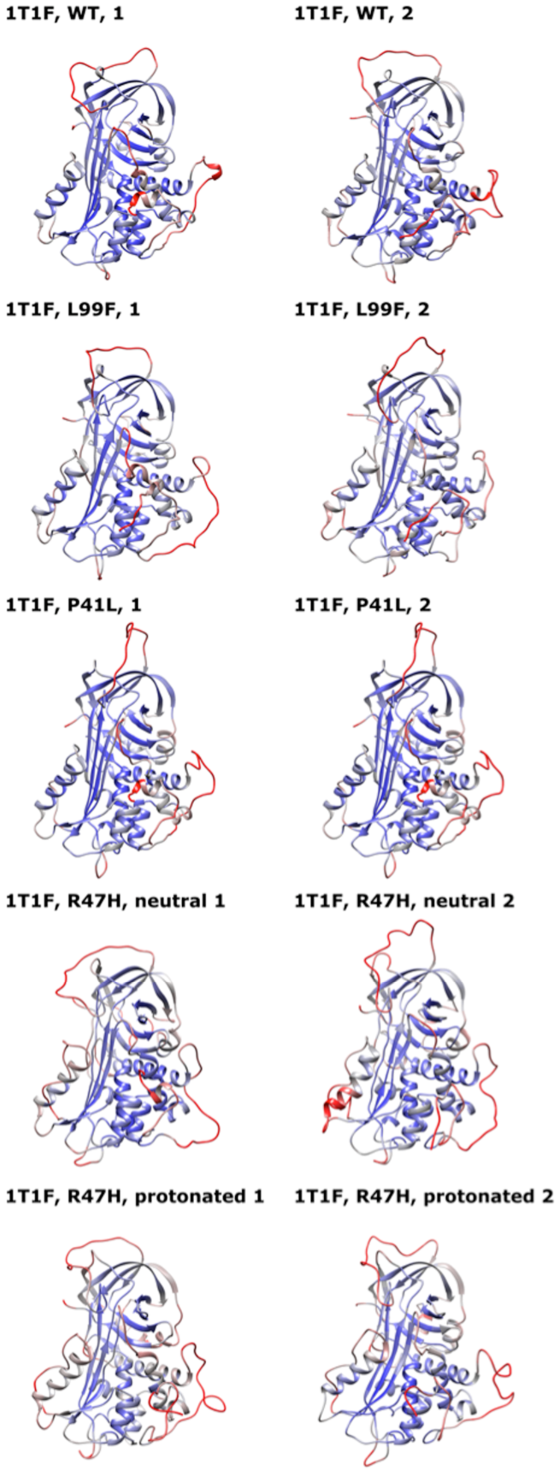

Supplementary figure 5: Root mean square fluctuations (RMSF) of the α-carbon atoms in the simulations not containing a pentasaccharide ligand ("1T1F-based"). The "ribbon" in the 3D models was colored according to the RMSF value, blue represents low, red corresponds to high fluctuations.

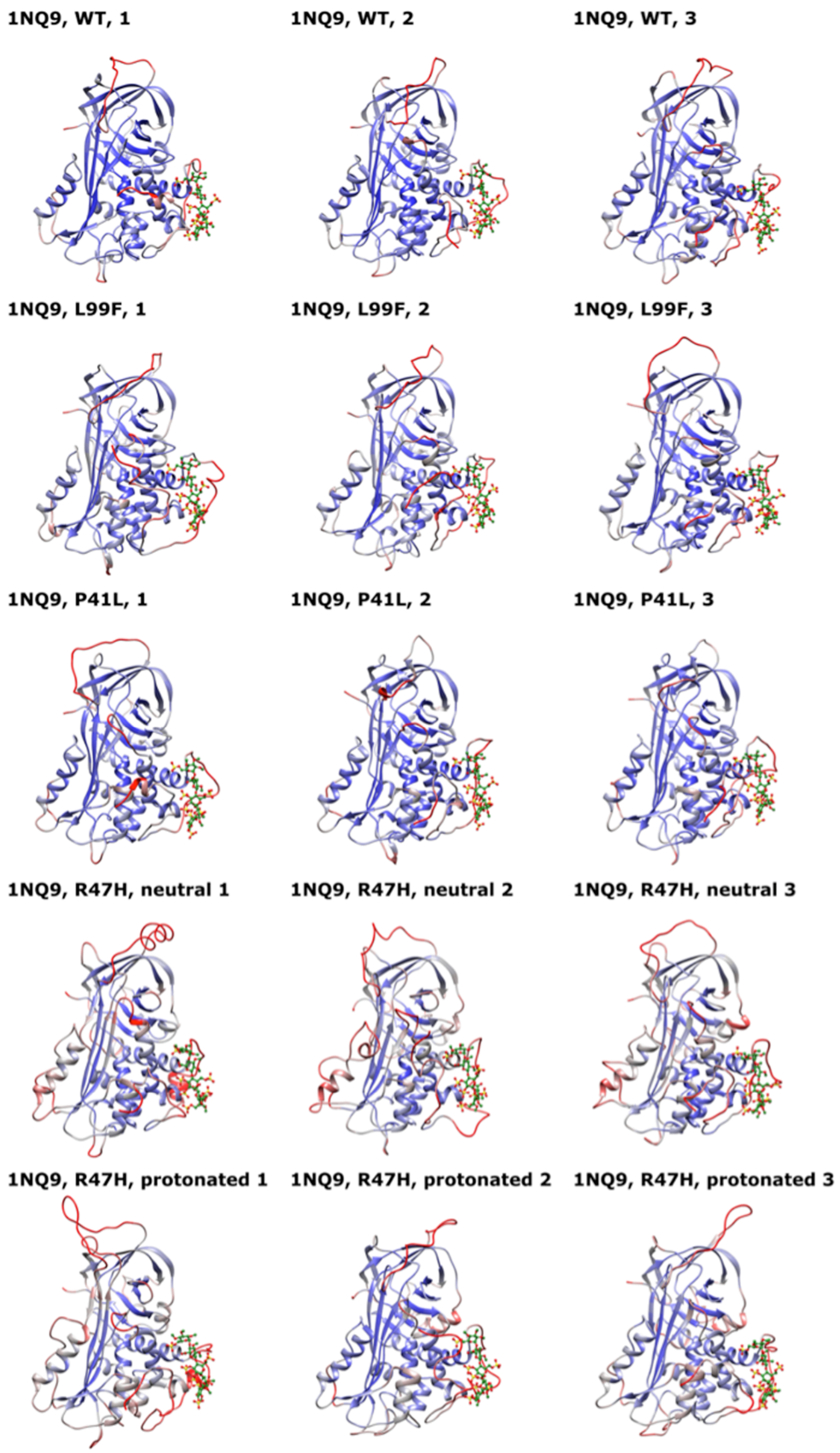

Supplementary figure 6: Root mean square fluctuations (RMSF) of the α-carbon atoms in the simulations of AT-pentasaccharide complex ("1NQ9-based"). The "ribbon" in the 3D models was colored according to the RMSF value, blue represents low, red corresponds to high fluctuations.

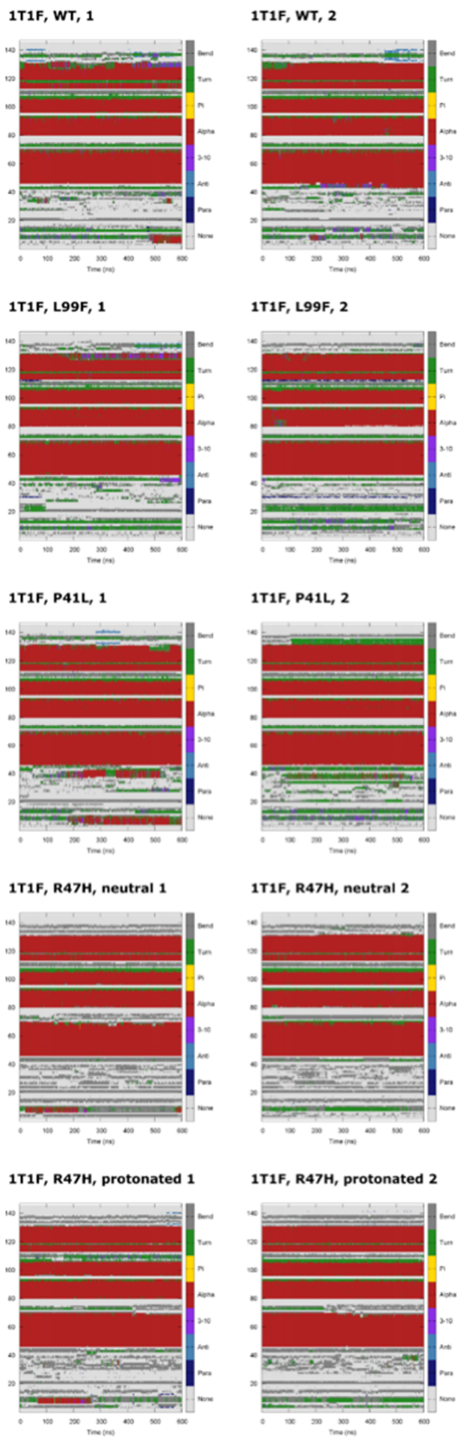

Supplementary Figure 7: Analysis of the secondary structure in region 1-145 (which includes helices P and D) using the DSSP method, from the simulations not containing the pentasaccharide ligand. (Para: Parallel β-sheet, Anti: Anti-parallel β-sheet, 3-10: 3-10 helix, Alpha: α-helix, Pi: Pi helix.)

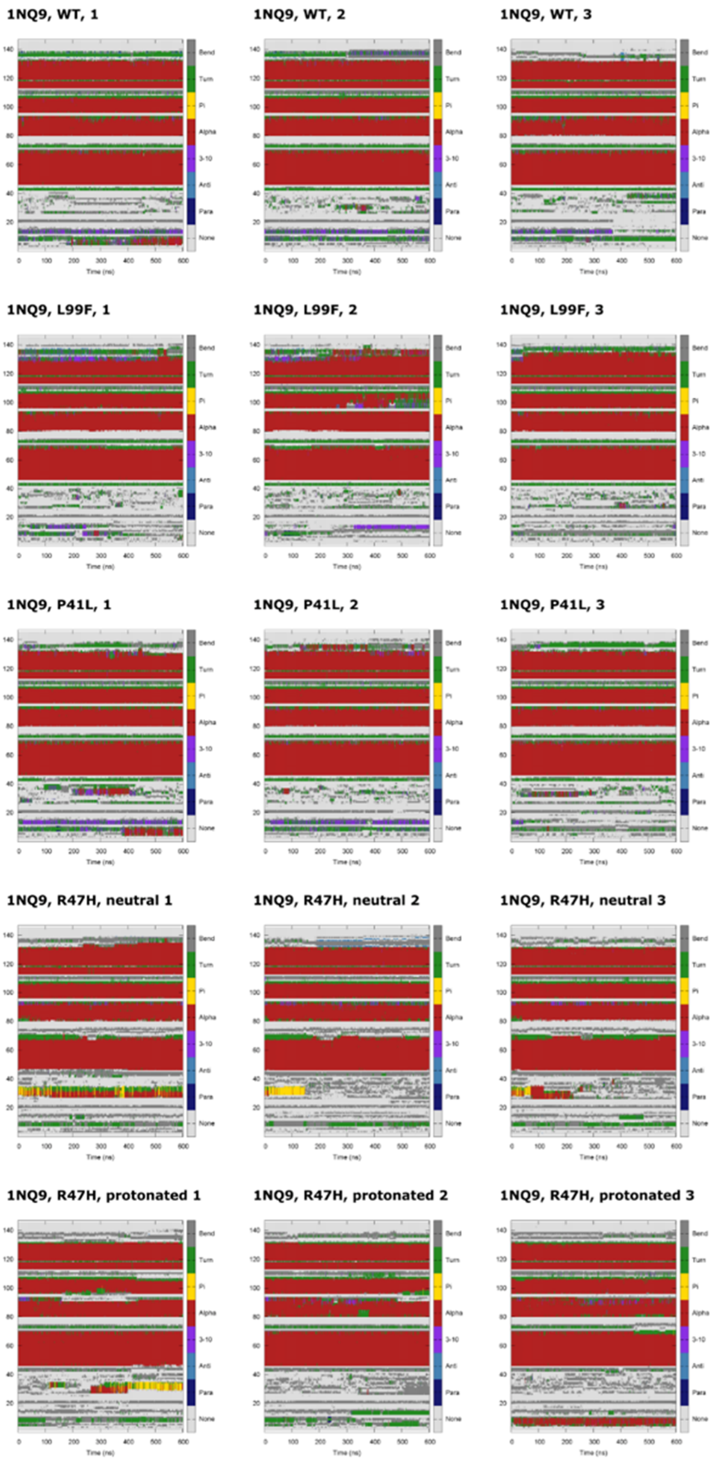

Supplementary Figure 8: Analysis of the secondary structure in region 1-145 (which includes helices P and D) using the DSSP method, from the AT-pentasaccharide complex simulations. (Para: Parallel β-sheet, Anti: Anti-parallel β-sheet, 3-10: 3-10 helix, Alpha: α-helix, Pi: Pi helix.)

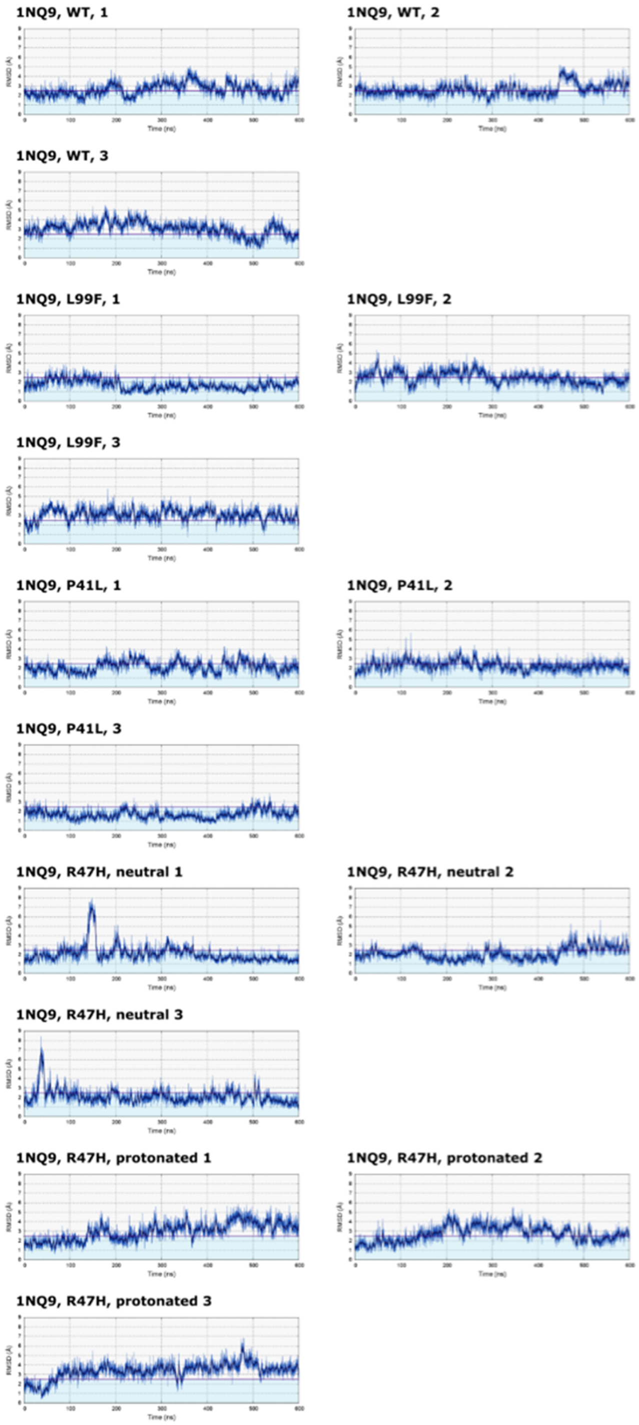

Supplementary figure 9: RMSD of the ring and interglycosidic atoms in the heparin pentasaccharide from all AT-pentasaccharide system simulations, compared to the X-ray diffraction structure 1NQ9.
